# The Effectiveness of a Chatbot Single-Session Intervention for People on Waitlists for Eating Disorder Treatment: Randomized Controlled Trial

**DOI:** 10.2196/70874

**Published:** 2025-05-21

**Authors:** Gemma Sharp, Bronwyn Dwyer, Alisha Randhawa, Isabella McGrath, Hao Hu

**Affiliations:** 1 Department of Neuroscience Monash University Melbourne Australia; 2 School of Psychology University of Queensland St Lucia Australia

**Keywords:** eating disorder, single-session intervention, chatbot, conversational agent, artificial intelligence, AI, mental health, digital health, randomized controlled trial, cognitive behavioral therapy, enhanced cognitive behavioral therapy

## Abstract

**Background:**

Early treatment is critical for improving eating disorder prognosis. Single-session interventions (SSIs) can provide short-term support to people on waitlists for eating disorder treatment; however, it is not always possible to access SSIs. We co-designed and developed a rule-based chatbot called ED ESSI (Eating Disorder Electronic Single-Session Intervention), which delivered an SSI and demonstrated its acceptability and feasibility. However, the effectiveness of ED ESSI is yet to be investigated.

**Objective:**

This study aimed to investigate the effectiveness of an SSI delivered by ED ESSI. We examined the chatbot’s impact on eating disorder pathology, psychosocial impairment, depression, anxiety, stress, and motivation for change or treatment in individuals aged ≥16 years on waitlists for treatment for all types of eating disorders.

**Methods:**

This multicenter 2-armed randomized controlled trial included 60 people on waitlists for eating disorder treatment in the chatbot intervention group (n=30, 50%) or the control group (n=30, 50%). The ED ESSI chatbot guided participants through a 30-minute SSI of assessment and psychoeducation, while the control group received web-based information on the same core topics covered in the SSI. There were 4 time points: before intervention or baseline (time point 1 [T1]), after intervention within 72 hours of baseline (T2), 1 month after baseline (T3), and 3 months after baseline (T4). Eating disorder pathology (primary outcome) and psychosocial impairment, depression, anxiety, and stress (secondary outcomes) were measured at T1, T3, and T4, and motivation for change or treatment (secondary outcome) was measured at all 4 time points. Furthermore, the System Usability Scale was implemented at T2 for the chatbot intervention group only.

**Results:**

ED ESSI induced significantly greater reductions compared to the control group in the primary outcome of eating disorder pathology (*P*=.003) and secondary outcomes of psychosocial impairment (*P*=.008), depression (*P*=.002), and anxiety (*P*=.040) over the 1- and 3-month time points, with small to moderate effects (Cohen *d*=0.15-0.53). Chatbot use also induced an increase in participants’ confidence in their ability to change (secondary outcome) immediately after use (T2), with a moderate effect (*P*<.001; Cohen *d*=0.74). The chatbot was rated as “excellent” in terms of usability. A significantly higher proportion of participants in the chatbot group (28/30, 93%) entered treatment by 3 months upon the opportunity being offered to them, compared to the control group, with a moderate effect (21/30, 70%; *P*=.042; ϕ=0.30).

**Conclusions:**

ED ESSI promptly induced improvements in eating disorder pathology, psychosocial impairment, depression, and anxiety, which were detectable at 1 month and maintained to 3 months. ED ESSI potentially represents an effective, accessible, and scalable form of early intervention for people aged ≥16 years waiting for eating disorder treatment. Further research is needed to determine the longer-term effectiveness of ED ESSI.

**Trial Registration:**

Australian New Zealand Clinical Trial Registry ACTRN12623000680662; https://tinyurl.com/2h9v7hh7

## Introduction

### Background

Eating disorders are complex conditions characterized by a disturbance in eating behavior that interferes with an individual’s physical and psychosocial functioning [1]. They are a global concern associated with a high fiscal and health burden. Due to their complexity, managing medical and psychiatric risks is an essential part of treatment [2]. Early intervention is considered best practice and is critical for improving eating disorder prognosis [3-5]. There is a well-documented risk of symptom exacerbation as an eating disorder progresses [6-9]. Moreover, research has indicated that more time spent on treatment waitlists is linked to a greater likelihood of treatment discontinuation [10,11]. Thus, interim interventions are urgently required to support people seeking treatment.

Single-session interventions (SSIs) have been proposed as a strategy to provide short-term support for those on waitlists for mental health treatment [12,13]. SSIs typically involve a one-off interaction with a health professional, structured to support individuals in making intentional, positive changes to their mental health [14]. SSIs have helped improve diverse mental health–related outcomes. In a meta-analysis of 50 randomized controlled trials (RCTs), SSIs for youth mental health problems (anxiety, depression, conduct problems, and general distress) demonstrated a significant beneficial effect compared to control conditions, reflecting a small-to-medium overall effect with positive impacts lasting up to approximately 13 weeks or 3 months [15]. For SSIs specifically focused on supporting people experiencing eating disorders, the literature is still emerging; however, SSIs are considered a promising strategy [13,16,17]. For example, preliminary research using a psychoeducation-focused, psychologist-delivered SSI for adults on waitlists for outpatient eating disorder treatment has demonstrated promising results, including reductions in eating disorder symptoms and clinical impairment [18]. While SSIs are traditionally conducted face-to-face, online SSIs have gained traction due to their accessibility, cost-effectiveness, and convenience, demonstrating levels of efficacy similar to face-to-face SSIs [14,15]. For example, a single-session consultation delivered via telehealth by a clinician for individuals on waitlists for outpatient psychotherapy was found to be acceptable, feasible, and effective in the short term, similar to the in-person offering [19]. In addition, this telehealth consultation elicited pre- to postintervention improvements in hopelessness and readiness for change and, at a 2-week follow-up, improvements in anxiety symptoms [19].

Conversational artificial intelligence agents or “chatbots” reflect a unique opportunity for online or digital service provision, and a growing body of research has indicated that chatbots have been positively received in the broader mental health care sector [20-22]. Chatbots offer several benefits, including wider accessibility, instantaneous responses, and low or no cost to users, and can act as a stepping stone to more individualized services [23,24]. Additional barriers to accessing traditional services can include mental health stigma or the lack of services available at the time, where individuals may feel uncomfortable or unable to seek the support they need [23,24]. A recent systematic review and meta-analysis of 18 RCTs evaluated the efficacy of psychotherapy-based chatbots, primarily using cognitive behavioral therapy (CBT) techniques, in alleviating depression and anxiety symptoms among adults. Findings indicated that interaction with the chatbots led to significant reductions in symptoms of both anxiety and depression, with small effect sizes observed [25]. However, it should be noted that all mental health chatbots included in the analysis delivered support over multiple weeks rather than an SSI [25].

Regarding eating disorders, only 4 chatbots that focused on eating disorders and body image have been reported in the literature to our knowledge: Tessa [26]*,* Topity [27], Alex [28], and KIT [23] (subsequently named JEM). Tessa, which was designed to deliver an 8-session CBT-based program, successfully reduced young women’s concerns about weight and shape in an RCT setting and has shown potential to reduce eating disorder onset [29]. However, there were serious concerns about the safety of this chatbot when it was modified for wider public use [30]. More specifically, in June 2023, Tessa chatbot deviated from its preprogrammed answers to provide dieting and weight loss advice, which can be particularly harmful in eating disorder settings. The incident received a great deal of publicity and commentary, and Tessa chatbot has not been implemented in any public setting since to our knowledge [31]. Before the June 2023 incident, Tessa was adapted into a “single session mini-course” that prompted significant improvements in users’ body image and motivation to change body image in a non-RCT setting [32]. Topity, which was created to deliver body image microinterventions, elicited small, notable improvements in state- and trait-based outcomes for body image and associated well-being constructs in an RCT involving adolescents [27]. Components of Alex*,* designed to target an individual’s motivation to engage with treatment, have been investigated in an RCT; however, the optimized version of the chatbot is still to be tested [33]. KIT/JEM was co-designed to provide body image– and eating disorder-focused psychoeducation and microinterventions to adolescents and young adults and their loved ones, leading to improvements in users’ body image and mood in naturalistic settings in multiple countries [34,35]; however, an RCT has not been conducted.

All these chatbots offer support for individuals with body image concerns and eating disorder pathology. However, these 4 conversational agents were not necessarily originally designed with a specific SSI focus. Moreover, they all address prehelp seeking or connecting users with help services rather than bridging a critical gap by providing timely assistance to treatment seekers who are on waitlists for services.

To overcome these limitations, we have developed, to our knowledge, a world-first chatbot, called ED ESSI (Eating Disorder Electronic Single-Session Intervention), a rule-based agent designed to deliver an SSI to older adolescents and adults on waitlists for eating disorder treatment. The co-design and co-development process of this chatbot was extensive, involving people who had recovered from an eating disorder and health professionals in the field of eating disorders [36]. This 30-minute SSI followed an enhanced-CBT (CBT-E) approach, similar to in-person SSI offerings for eating disorders [18], and focused on psychoeducation and assessment for all types of eating disorders. The broad goals of ED ESSI were to promote improvements in eating disorder and other psychological well-being symptoms while people were waiting for treatment as well as increase motivation for engaging with in-person treatment once it became available. In the co-design process, although there were initial concerns from recovered participants and health professionals regarding the chatbot’s ability to provide appropriate empathy and validation as well as monitor medical and psychiatric risk, strategies were co-designed and implemented [36]. Our co-design participants deemed a rule-based approach, where the chatbot could only deliver preprogrammed responses, as the safest strategy. The co-design process also involved reports that seeking support for eating disorder care was highly challenging, given the stigma surrounding eating disorders in particular, so offering a nonhuman alternative that still had an interactive chat interface with a range of activities was seen as optimally engaging [36]. The evaluation of the final prototype version of ED ESSI was positive from people with a lived experience of an eating disorder and health professionals [36].

### Objectives

Although preliminary feasibility and acceptability for ED ESSI have been demonstrated [36], we are yet to determine the chatbot’s effectiveness in an RCT and, in addition, in real-world clinical waitlist settings. Hence, we aimed to conduct the first RCT to determine the effectiveness of ED ESSI for people aged ≥16 years on the waitlist for eating disorder treatment at outpatient psychology clinics around Australia. It was hypothesized that participants who engaged with the chatbot SSI would experience greater reductions in their eating disorder pathology (primary outcome) after 1 and 3 months compared to a control group that received similar web-based psychoeducation information but not delivered by a chatbot in the form of an SSI. Furthermore, it was predicted that the SSI chatbot use would elicit greater improvements in broader facets of well-being (secondary outcomes), specifically psychosocial impairment due to eating disorder symptoms, mood (depression, anxiety, and stress), and motivation for treatment after 1 and 3 months compared to the control group.

## Methods

### Study Design

This study was a 2-arm, parallel-assignment, and nonblinded RCT with an intervention group and a control group. Screening at least 70 participants (allowing for 15% dropout [37]) would allow for the detection of small to moderate Cohen *f* effect size changes (Cohen *f*=0.17) in eating disorder pathology (primary outcome) between groups, in accordance with findings from Fursland et al [18], with a power of 0.80 and a significance level of *P*=.05 [38]. Simple 1:1 randomization was performed using a Python (Python Software Foundation) script. Allocation was not concealed, allowing investigators to assign participants to either group based on the randomization result.

### Ethical Considerations

The study was prospectively registered on the Australian New Zealand Clinical Trials Registry (ACTRN12623000680662) [39] and approved by the Monash University Human Research Ethics Committee (ID 38277). Participants provided written informed consent to be enrolled in the study via a web-based survey through Qualtrics (Qualtrics International Inc). Participants were provided with the ethics-approved study information by the recruiting clinics immediately upon joining the clinic’s waitlist, and those aged 16 to 17 years were considered sufficiently mature to provide consent for themselves under our ethics approval. Participants were assigned a unique code to include in each of the 4 time point surveys to deidentify their responses. All participants were reimbursed after the final study time point with an Aus $50 (US $32.36) voucher.

### Participants

Participants were recruited from outpatient psychology clinics or practices around Australia from September 2023 to September 2024. Participants had been referred by their general practitioners or primary care physicians before seeking care from these clinics or practices. All clinics involved charged fees for their treatment services, which is very common for eating disorder treatment in Australia [40], but no fees were charged to participants for study involvement. Participant inclusion criteria were (1) being aged ≥16 years and (2) being registered on an outpatient psychology clinic waitlist to commence eating disorder treatment following a referral from a general practitioner or primary care physician. Exclusion criteria were (1) the lack of access to a working email address or electronic device and (2) high medical and/or psychiatric risk, as assessed and documented by a general practitioner or primary care physician as a standard part of referral to an outpatient psychology clinic for eating disorder treatment. There was 1 deviation in the inclusion or exclusion criteria for the preregistered protocol [39], namely, participants with any eating disorder diagnosis were eligible.

After the provision of informed consent and study enrollment (refer to Ethical Considerations section), the research team members performed the randomization, and participants were informed of the group to which they had been allocated—the chatbot intervention or the control group. In an attempt to reduce the effect of expectation bias, the research team intentionally masked our predictions that the chatbot would elicit greater clinical outcome improvements compared to the control. The research team members remained unblinded, in addition to the participants. The uptake of the chatbot intervention or control information was monitored via email, with the participants confirming completion via email to the research team. Furthermore, we were able to confirm the participant’s use of the chatbot through their log-in history in the chatbot web portal.

### Intervention

#### ED ESSI Chatbot Intervention Group

**Figure 1:**
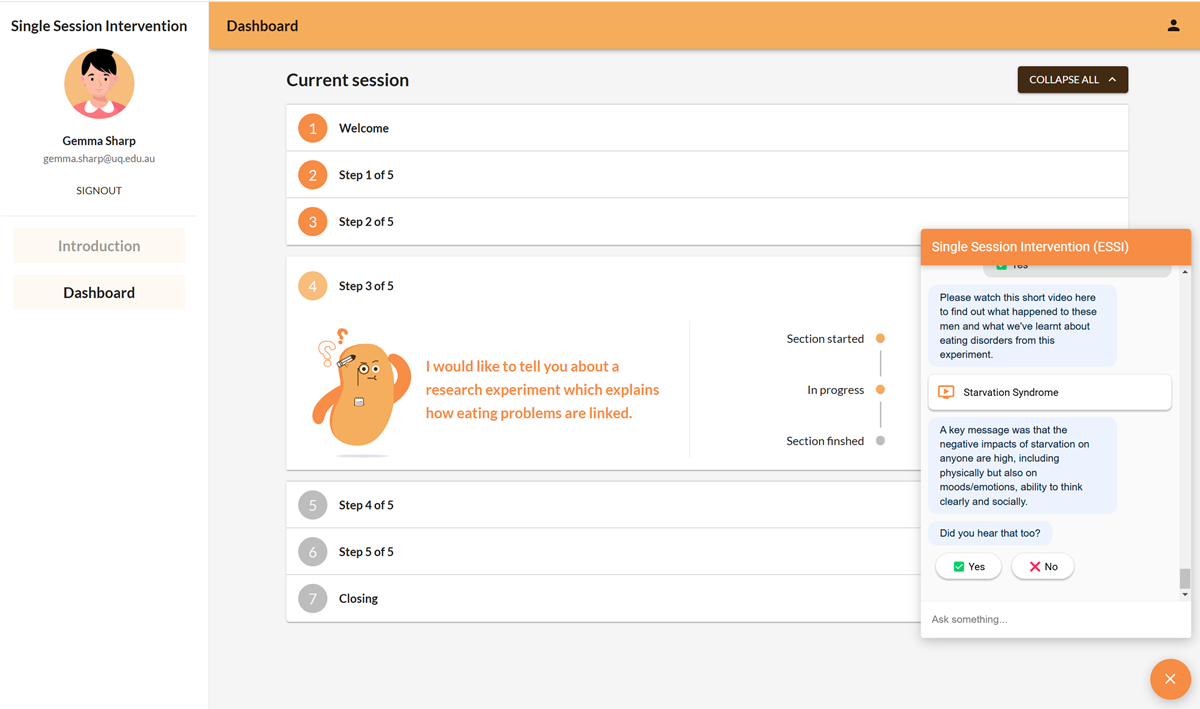
An example of an ED ESSI (Eating Disorder Electronic Single-Session Intervention) conversation providing psychoeducation on starvation syndrome via conversation and video.

Participants in the intervention group were given access (via email and password-protected log-in) to a 30-minute SSI delivered by our web-based and rule-based chatbot ED ESSI (refer to the study by Sharp et al [36] for a detailed description of the intervention and Figure 1). This SSI was broadly adapted from the psychologist-delivered SSI framework proposed by Fursland et al [18] and followed a CBT-E approach. It involved a conversation, short videos, and interactive activities focused on symptom assessment and psychoeducation about starvation syndrome, the maintenance cycle of eating disorders, and regular eating for recovery [36].

#### Control Group

Participants in the control group were emailed web links by the researchers to brief, publicly accessible psychoeducation information sheets addressing the same major topics discussed in the chatbot SSI, specifically starvation syndrome, the vicious cycles of dieting, and regular eating to promote recovery [41]. These information sheets took around 30 minutes to read. It should be noted that, while not a deviation from the preregistered protocol [39], we clarify that the “typical treatment for waitlist clients, which is no treatment” we described in the protocol usually includes patients being provided with a range of online information while waiting. Thus, we simulated a standard outpatient waitlist experience with our control group.

### Outcome Assessment

#### Assessment Time Points

There were four assessment time points: (1) at baseline or study enrollment (time point 1 [T1]), (2) within 72 hours of baseline and immediately after the completion of the chatbot or control intervention (T2), (3) 1 month after baseline (T3), and (4) 3 months after baseline (T4).

#### Overview

All assessments were administered via web-based surveys through Qualtrics. Participants were assigned a unique code to include in each survey to deidentify their responses across time points. At baseline, participants were asked demographic and clinical characteristic questions (age, gender, height, weight, ethnicity, educational attainment, eating disorder diagnosis as indicated in their initial referral, other mental health diagnoses, and previous eating disorder treatment) in addition to the primary and secondary outcome measures (described subsequently). In addition, at T3 and T4, participants were asked whether they had started treatment, that is, whether they were no longer on the waitlist. If they had not started treatment, they were asked to explain the reasoning via an open-ended textbox. If they had started treatment, they were asked to state the number of appointments with a psychologist they had attended.

#### Eating Disorder Pathology

The change in eating disorder symptoms (primary outcome) was assessed at T1, T3, and T4 using the Eating Disorder Examination Questionnaire (EDE-Q) [42], which comprised 28 self-report items that contributed to 4 subscales. Notably, this questionnaire was administered at T1, T3, and T4 as these time points were >28 days apart, the time frame used in the questionnaire. The 4 subscales of the EDE-Q measured dietary restraint (5 items; eg, “Have you tried to limit the amount of food you eat?”), eating concern (5 items; eg, “Have you felt guilty after eating?”), shape concern (8 items; eg, “Has your shape influenced how you think about yourself as a person?”), and weight concern (5 items; eg, “Have you been dissatisfied with your weight?”). Responses reflected the past 28 days, using a scale from 0 (“no days”) to 6 (“every day”) or from 0 (“not at all”) to 6 (“markedly”). Subscale scores were calculated as the mean of the items within each subscale, and the global score was the mean of all subscale scores, ranging from 0 to 6. Higher scores indicated greater severity of eating disorder pathology. A global score of ≥3.0 is part of the eligibility criteria to access specialized outpatient eating disorder treatment plans in Australia [43]. The EDE-Q global scale demonstrated high reliability in this study’s sample (Cronbach α at T1=0.90, T3=0.95, and T4=0.93) and all the subscales: dietary restraint (Cronbach α at T1=0.76, T3=0.86, and T4=0.77), eating concern (Cronbach α at T1=0.70, T3=0.79, and T4=0.73), shape concern (Cronbach α at T1=0.80, T3=0.91, and T4=0.91), and weight concern (Cronbach α at T1=0.74, T3=0.83, and T4=0.74).

#### Psychosocial Impairment

The change in the severity of psychosocial impairment (secondary outcome) due to eating disorder symptoms was assessed at T1, T3, and T4 (given the questionnaire’s 28-day time frame) using the Clinical Impairment Assessment (CIA) [44]. This measure comprised 16 self-report items across 3 subscales: personal impairment (6 items; eg, “To what extent have your eating habits, exercising, or feelings about your eating, shape or weight made you feel upset?”), cognitive impairment (5 items; eg, “To what extent have your eating habits, exercising, or feelings about your eating, shape or weight made it hard to concentrate?”), and social impairment (5 items; eg, “To what extent have your eating habits, exercising, or feelings about your eating, shape or weight stopped you from socialising?”). Respondents rated each item on a 4-point Likert scale ranging from 0 (“not at all”) to 3 (“a lot”), with higher scores indicating greater functional impairment. The total score was derived from the sum of all item scores, which could range from 0 to 48, with a score of ≥16 representing clinically significant impairment [44]. The CIA global scale demonstrated high reliability in this study’s sample (Cronbach α at T1=0.94, T3=0.94, and T4=0.95) and the subscales: personal impairment (Cronbach α at T1=0.89, T3=0.91, and T4=0.90), cognitive impairment (Cronbach α at T1=0.93, T3=0.88, and T4=0.89), and social impairment (Cronbach α at T1=0.88, T3=0.91, and T4=0.92).

#### Depression, Anxiety, and Stress

Changes in depression, anxiety, and stress (secondary outcomes) were assessed at T1, T3, and T4 using the Depression Anxiety and Stress Scale-21 (DASS-21) [45], which comprised 21 items, with 7 items contributing to each of the 3 subscales: depression, anxiety, and stress. To complete the DASS-21, respondents rated the extent to which each statement (eg, “I felt sad and depressed” [depression]; “I felt scared for no reason” [anxiety]; “I found it hard to relax” [stress]) applied to them over the past week on a 4-point Likert scale, ranging from 0 (“Did not apply to me at all”) to 3 (“Applied to me very much, or most of the time”). Subscale scores were calculated as the sum of the relevant items and multiplied by 2 to align with the original Depression Anxiety and Stress Scale-42 scoring system [45], with subscale scores ranging from 0 to 42. Higher scores indicated greater severity of symptoms in the respective domains. Scores are typically categorized into “normal,” “mild,” “moderate,” “severe,” and “extremely severe” categories [45]. The DASS-21 depression subscale demonstrated high reliability in this study sample (Cronbach α at T1=0.92, T3=0.94, and T4=0.95) as well as the anxiety subscale (Cronbach α at T1=0.87, T3=0.88, and T4=0.83) and stress subscale (Cronbach α at T1=0.71, T3=0.84, and T4=0.85).

#### Motivation for Treatment

At all 4 time points, motivation for treatment was assessed using visual analog scales adapted from Schmidt et al [46]. Participants answered the following questions on an 11-point scale, ranging from 0 (“not at all”) to 10 (“very much”): (1) “How important is it for you to change?” (2) “How confident are you in your ability to change?” and (3) “How comfortable do you feel about your upcoming treatment?” The 3 items were investigated separately, and higher scores indicated higher motivation for treatment.

#### System Usability

At T2, only the participants in the chatbot intervention group completed the System Usability Scale (SUS) [47] immediately after use, which comprised 10 items that reflected various statements related to system usability (eg, “I thought the system was easy to use”). Responses were recorded on a 5-point Likert scale ranging from 1 (“strongly disagree”) to 5 (“strongly agree”). The SUS score yielded a single number between 0 and 100, with higher scores representing higher perceived usability of the system. The SUS demonstrated high reliability in this study’s sample (Cronbach α=0.83). In addition to the SUS, we asked participants to provide any extra feedback they had about using the chatbot via 1 item with an open-ended text format.

### Data Analysis

SPSS (version 30; IBM Corp) [48] was used for quantitative statistical analysis. Descriptive statistics were used to analyze demographic and clinical characteristics. To determine whether there were differences between groups at baseline (or other time points as required), independent 2-tailed *t* tests were used for continuous data and Fisher exact test for categorical data. To examine the effects of the chatbot versus control intervention across the 3 or 4 time points on the primary and secondary outcomes, mixed ANOVA with the intervention group as the between factor and time as the within factor were conducted with partial eta squared as the overall effect size. Significant group×time interaction effects were further analyzed using paired sample 2-tailed *t* tests, with a conservative Bonferroni correction applied to mitigate against the increased risk of a type I error for the multiple pairwise tests [49] and Cohen *d* used as the effect size [50]. Any missing data were handled with listwise deletion. The open-ended qualitative text responses addressing chatbot feedback and reasons for not commencing treatment were analyzed using an abbreviated content analysis approach [51], given the brevity of participant responses (30 words maximum).

## Results

### Participants

**Figure 2:**
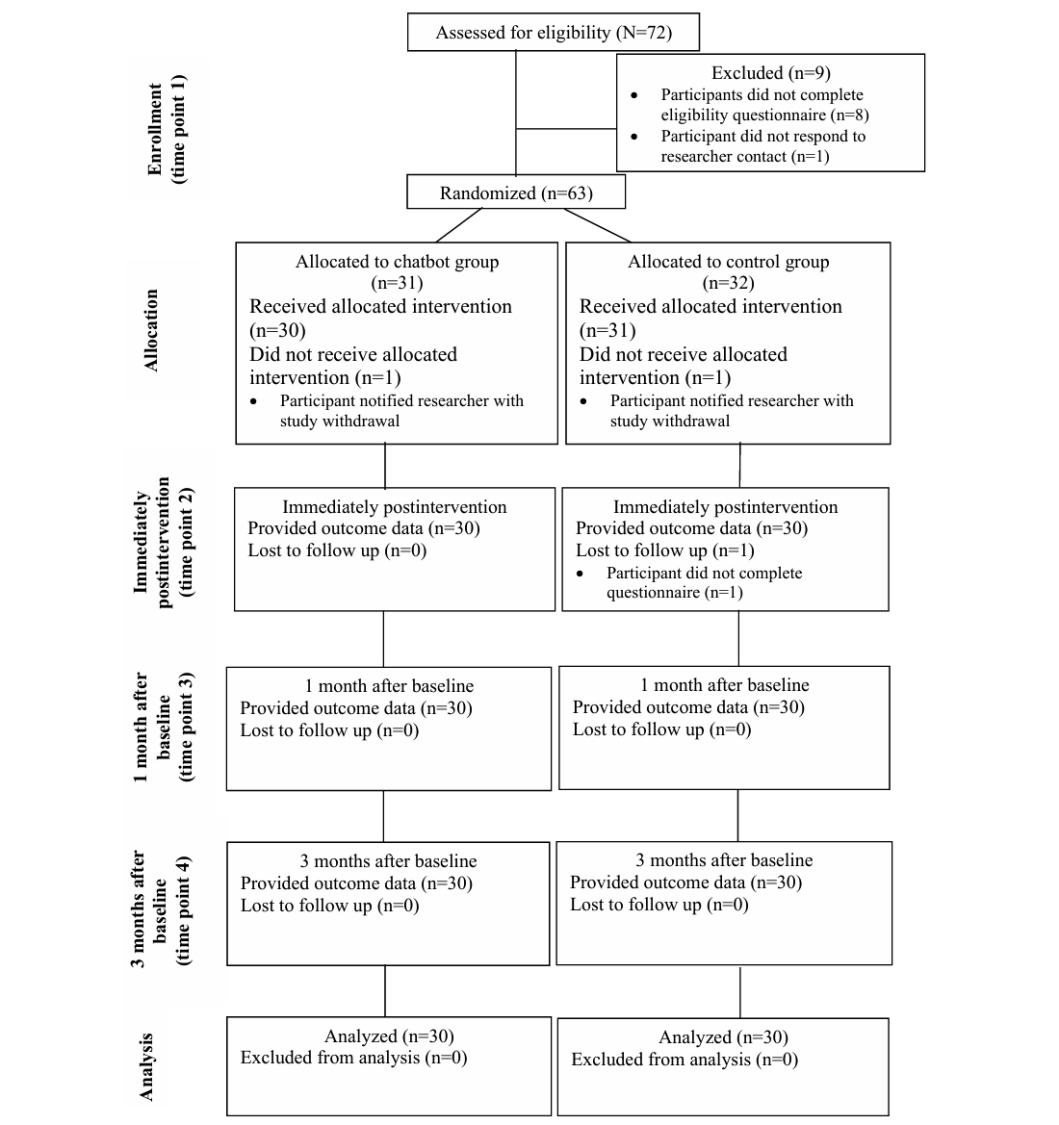
Overview of participant flow through stages of the randomized controlled trial.

Of the 72 people screened for eligibility, 63 (88%) were admitted and 60 (83%) ultimately completed the study (refer to Figure 2 for an overview of the participant flow). Demographic characteristics at T1 or baseline are provided in Table 1, where there were no significant differences between the chatbot and control groups for any of the characteristics measured (all *P*>.05).

**Table 1:** Participant demographic characteristics by group at baseline (time point 1; N=60).

Characteristics		Chatbot (n=30)	Control (n=30)
Age (y), mean (SD; range)		30.6 (11.8; 16-58)	31.1 (11.8; 16-60)
**Gender, n (%)**			
	Woman	26 (87)	24 (80)
	Man	2 (7)	3 (10)
	Gender diverse	2 (7)	3 (10)
**Ethnicity, n (%)**			
	Asian	2 (7)	2 (7)
	White	27 (90)	26 (87)
	Mixed	1 (3)	2 (7)
**Education, n (%)**			
	Below high school completion	4 (13)	2 (7)
	High school completion or equivalent	9 (30)	11 (37)
	Vocational qualification	1 (3)	1 (3)
	Diploma	3 (10)	3 (10)
	Postgraduate diploma	0 (0)	1 (3)
	Bachelor’s degree	10 (33)	8 (27)
	Master’s degree	3 (10)	3 (10)
	Doctorate	0 (0)	1 (3)
**Eating disorder diagnosis, n (%)**			
	Other specified feeding or eating disorder	12 (40)	10 (33)
	Bulimia nervosa	9 (30)	8 (27)
	Anorexia nervosa	5 (17)	5 (17)
	Binge eating disorder	2 (7)	4 (13)
	Avoidant/restrictive food intake disorder	2 (7)	3 (10)
BMI (kg/m^2^), mean (SD; range)		25.8 (10.5; 13.2-60.4)	26.1 (10.8; 14.0-57.0)
Mental health diagnosis besides eating disorder, n (%)		21 (70)	20 (67)
Previous treatment received for eating disorder, n (%)		17 (57)	15 (50)

### Primary and Secondary Outcomes

The descriptive statistics for the chatbot and control groups over the time points are outlined in Tables 2 and 3 (refer to Multimedia Appendix 1 for all EDE-Q and CIA subscale descriptive statistics). There were no significant differences between the chatbot and control groups for any of the measure outcomes at T1 or baseline (all *P*>.05). At baseline, the participant scores were (on average and as expected) well above the EDE-Q global score required to meet eligibility for specialized eating disorder treatment plans in Australia. According to the mean CIA global score, the participants, on average, met the threshold for clinically significant impairments in their lives owing to eating disorder symptoms. The mean depression, anxiety, and stress symptoms were in the moderate, severe, and moderate ranges, respectively. Furthermore, in terms of motivation, the participants rated it highly important that they change, and scores were moderate for confidence in their ability to change and comfort toward treatment at baseline.

**Table 2:** Primary and secondary outcomes for the chatbot (n=30) versus control (n=30) intervention over the baseline (time point 1 [T1]), 1-month postbaseline (T3), and 3-months postbaseline (T4) time points.

Outcome measures			T1 score, mean (SD)		T3 score, mean (SD)		T4 score, mean (SD)
**EDE-Q^a^ global**							
	Chatbot	4.0 (1.2)		3.5 (1.3)		3.1 (1.2)	
	Control	4.0 (1.1)		3.9 (1.1)		3.7 (1.0)	
**CIA^b^ global**							
	Chatbot	33.6 (10.1)		27.9 (11.5)		23.6 (11.5)	
	Control	34.7 (10.6)		33.9 (9.6)		29.6 (8.0)	
**DASS^c^ depression**							
	Chatbot	19.0 (9.5)		17.4 (10.1)		15.4 (10.7)	
	Control	20.2 (9.6)		19.2 (8.7)		18.7 (8.0)	
**DASS anxiety**							
	Chatbot	16.8 (10.6)		13.1 (10.4)		11.6 (8.7)	
	Control	17.8 (10.6)		17.3 (10.0)		16.0 (7.0)	
**DASS stress**							
	Chatbot	21.6 (7.7)		20.0 (9.5)		18.8 (9.7)	
	Control	22.2 (7.4)		22.2 (7.3)		20.5 (6.1)	

^a^EDE-Q: Eating Disorder Examination Questionnaire.

^b^CIA: Clinical Impairment Assessment.

^c^DASS: Depression Anxiety and Stress Scale.

**Table 3:** Motivational outcomes for the chatbot (n=30) versus control (n=30) groups over the baseline (time point 1 [T1]), immediately postintervention (T2), 1-month postbaseline (T3), and 3-month postbaseline (T4) time points.

		T1 score, mean (SD)	T2 score, mean (SD)	T3 score, mean (SD)	T4 score, mean (SD)
**Importance**					
	Chatbot	8.8 (1.5)	9.1 (1.2)	8.8 (1.1)	8.7 (1.5)
	Control	8.8 (1.4)	9.0 (1.3)	8.8 (1.6)	8.9 (1.6)
**Confidence**					
	Chatbot	4.9 (2.4)	6.5 (1.6)	5.3 (2.8)	5.8 (2.7)
	Control	5.0 (2.3)	5.1 (1.6)	5.0 (1.8)	5.0 (1.7)
**Comfort**					
	Chatbot	5.2 (2.8)	6.3 (2.3)	5.3 (2.7)	5.9 (2.6)
	Control	5.0 (2.9)	5.1 (1.7)	4.9 (1.8)	4.9 (1.9)

### Eating Disorder Pathology

As predicted, there was a significant group×time effect for EDE-Q global scores (*F*_2,116_=5.98; *P*=.003; η_p_^2^=0.09). For the chatbot group, there was a significant reduction in eating disorder pathology from baseline to 1 month (*P*<.001; Cohen *d*=0.40) and another significant reduction from 1 month to 3 months (*P*=.018; Cohen *d*=0.29). At 3 months, the mean score had reduced such that it was only slightly above the eligibility criterion for specialized eating disorder treatment plans in Australia (≥3.0). For the control group, although there was a trend toward a small reduction in scores over the 3 months, these were not significant, unlike for the chatbot group (T1 vs T3: *P*=1.000; Cohen *d*=0.07 and T3 vs T4: *P*=.712; Cohen *d*=0.14).

### Psychosocial Impairment

In concordance with prediction, there was a significant group×time effect for CIA global scores (*F*_2,116_=4.90; *P*=.008; η_p_^2^=0.08). The psychosocial impairment scores for the chatbot group significantly reduced from baseline to 1 month (*P*<.001; Cohen *d*=0.53) and had another significant decrease from 1 to 3 months (*P*=.001; Cohen *d*=0.37). There was no significant change from baseline to 1 month for the control group (*P*=1.000; Cohen *d*=0.07) but a significant reduction from 1 month to 3 months (*P*<.001; Cohen *d*=0.35). The chatbot group’s CIA global scores at the 3-month postbaseline time point were still significantly lower than the control group as predicted (*P*=.023; Cohen *d*=0.61). Both groups remained in the clinically significant impairment range (mean score ≥16.0) at the final time point at 3 months.

### Depression

Unexpectedly, there was no significant group×time effect for the DASS depression subscale (*F*_2,116_=1.40; *P*=.251; η_p_^2^=0.02). However, the depression scores decreased for both groups from baseline to 3 months, and upon further investigation, only the chatbot intervention elicited a significant decrease (*P*=.002; Cohen *d*=0.35 and control: *P*=.382; Cohen *d*=0.16). The mean depression scores for both groups remained in the moderate range at 3 months.

### Anxiety

As expected, there was a significant group×time effect for the DASS anxiety subscale (*F*_2,116_=3.61; *P*=.040; η_p_^2^=0.06). The anxiety scores for the chatbot group significantly reduced from baseline to 1 month (*P*<.001; Cohen *d*=0.35) but no significant change from 1 to 3 months (*P*=.248; Cohen *d*=0.15). For the control group, although there was a trend toward a small reduction in scores over the 3 months, these were not significant (T1 vs T3: *P*=1.000; Cohen *d=*0.05 and T3 vs T4: *P*=.419; Cohen *d=*0.12). The mean anxiety score for the chatbot group was reduced to the moderate range by T4, while the control group remained in the severe range.

### Stress

Although it appeared that the scores for stress decreased for both groups, slightly more so for the chatbot group, there was neither a significant group×time effect (*F*_2,116_=0.56; *P*=.575; η_p_^2^=0.01) nor were there significant reductions for either group from baseline to 3 months (chatbot—T1 vs T3: *P*=.420; Cohen *d*=0.18 and T3 vs T4: *P*=.590; Cohen *d*=0.13; control—T1 vs T3: *P*=1.000; Cohen *d*=0.01 and T3 vs T4: *P*=.225; Cohen *d*=0.23). The mean scores for both groups remained in the moderate range at the final time point.

### Motivation for Change and Treatment

Participant-rated importance to change was highly ranked for both groups across all 4 time points; however, in contrast to prediction, there was no significant group×time effect (*F*_3,174_=0.29; *P*=.830; η_p_^2^=0.01) and no significant changes for either group across the time points (all *P*>.05). Nevertheless, there was a significant group×time effect for confidence to change (*F*_3,174_=3.46; *P*=.018; η_p_^2^=0.06). There was a significant increase in confidence for the chatbot group from T1 to T2 (immediately postchatbot use; *P*<.001; Cohen *d*=0.74) but a significant decrease from T2 to T3 (*P*<.001; Cohen *d*=0.48) and no change overall from T1 to T4 (*P*=.328; Cohen *d*=0.32), hence staying at a moderate level of confidence. There was no significant change in the confidence for the control group for all time points (all *P*>.05), remaining at a moderate level. Finally, for comfort toward treatment, while there (similarly) appeared to be an increase for the chatbot group from T1 to T2 and then a decrease, the group×time effect was not significant (*F*_3,174_=2.08; *P*=.105; η_p_^2^=0.04), and no significant changes were observed for either group across the time points (all *P*>.05). It appeared that the comfort toward treatment remained at a moderate level for both groups across the 4 time points.

### Chatbot Usability

The mean score for the SUS for the chatbot group was 83.8 (SD 12.5), which equated to an “excellent” or “A” grade category [47]. In total, 40% (12/30) of the participants used the opportunity to provide a very brief comment in the open-ended text option about their experience with the chatbot. These were predominantly positive comments about the “usefulness” and “helpfulness” of the chatbot (10/30, 33%). A small number of participants (3/30, 10%) reported that they would have appreciated the option to go backward in the chatbot conversation to change or clarify some of their earlier answers in the SSI. This backward function was not available in the ED ESSI version used in the RCT.

### Exploratory Findings

All participants were on a waitlist for treatment at T1 (baseline) and T2 (immediately after intervention), but participants were also asked whether they had commenced treatment at 1 month (T3) and 3 months (T4). If not, an opportunity to provide a very brief update on their treatment commencement was offered, and if they had started treatment, participants nominated how many treatment sessions they had completed.

At 1 month, 50% (15/30) of the participants in the chatbot group and 47% (14/30) of the participants in the control group had started treatment, so there were no significant differences between the groups according to Fisher exact test (*P*=1.000; ϕ=0.03). Among the chatbot group’s 15 participants who had not started treatment, 13 (87%) reported that they were still on the waitlist, and 2 (13%) reported that they had “rescheduled their first session” owing to a scheduling conflict. In the control group, 15 (94%) of the 16 participants reported that they were on the waitlist, and 1 (6%) participant reported that they had to reschedule their first session. For the participants who had started treatment, there was no significant difference between the number of treatment sessions attended for the chatbot (mean 2.3, SD 1.4) and control (mean 1.9, SD 0.7) groups (t_27_=0.81; *P*=.424; Cohen *d*=0.30).

At the 3-month postbaseline time point, 28 (93%) of the 30 participants had started treatment in the chatbot group, while only 21 (70%) of the 30 participants in the control group had started treatment, which represented a significant difference (*P*=.042; ϕ=0.30). The 2 participants in the chatbot group who had not started treatment clarified that they had been offered a first appointment, but “financial constraints” or “money concerns” meant that they had not pursued treatment. In the control group, similarly, all participants had been offered a first appointment, with all (9/9, 100%) participants stating that financial constraints or money concerns had prevented their engagement and 4 (44%) participants additionally mentioning “concerns” about their “confidence in [their] ability to recover.” For the participants engaged in treatment, similar to the 1-month postbaseline time point, there was no significant difference between the number of treatment sessions attended for the chatbot (mean 4.9, SD 2.4) and control (mean 4.3, SD 1.1) groups (t_35_=1.154; *P*=.256; Cohen *d*=0.32).

## Discussion

### Principal Findings

This study aimed to examine the effectiveness, for the first time, of a novel SSI chatbot, ED ESSI, for older adolescents and adults on the waitlist for outpatient eating disorder treatment. Through an RCT in clinical settings, we demonstrated that chatbot use induced early reductions in eating disorder pathology (primary outcome) and psychosocial impairment due to eating disorder symptoms (secondary outcome) at 1 month and then further reductions after that time to 3 months with small to moderate effects. The control group who received similar information as in the chatbot SSI group did not show these decreases, although there was a small, significant reduction in psychosocial impairment by 3 months in the control group.

For the other (secondary) outcomes, the chatbot-induced early anxiety reductions were detected at 1 month, such that symptoms were classified in the moderate range (reduced from severe), and depression symptoms decreased by 3 months (moderate range); however, there was no change in stress symptoms (moderate range). There were no changes in the mood measures for the control group. Unexpectedly, there were minimal differences between the chatbot and control groups for measures of motivation. There was a moderate to large increase in confidence for the chatbot group immediately after chatbot use, but this was not maintained. Nevertheless, these ratings were in the moderate to high range for both groups at the start of the RCT, so there was less opportunity for improvement across the trial.

Overall, it appeared that the experience with ED ESSI promoted early gains (not evident in the control group) that were able to be leveraged once the individual left the waitlist and started treatment with a psychologist. The results suggested that those who used the chatbot were significantly more likely to start treatment by 3 months, possibly because of the early improvements they experienced. Importantly, chatbot users were still in the clinical symptom and psychosocial impairment ranges by the 3-month postbaseline time point, although much closer to entering subclinical ranges. Nevertheless, it was not the intention that a 30-minute SSI delivered by a chatbot would alleviate all symptoms; rather, it would provide individuals with a “head start” on treatment and assist with the transition to in-person treatment, which is recommended to be between 20 and 40 sessions with a psychologist for CBT-E [42]. Even though it seems like waitlist lengths for eating disorders peaked during the COVID-19 pandemic [52] and have subsequently reduced, positive and effective early treatment experiences remain crucial to implement, and ED ESSI is a promising, convenient, and accessible strategy.

### Comparison With Prior Work

ED ESSI chatbot is an important addition to the short but emerging list of chatbots focused on eating disorders and/or body image (and mental health chatbots in general) that have been found to induce small to moderate improvements in the mental health of users [25,27,29,32,33]. ED ESSI was able to induce these improvements in a novel way, more specifically, in a clinical sample and in clinical settings rather than community-based studies. We are seemingly still relying on simpler rule-based conversational agents in the field of eating disorders, most likely owing to the high level of medical and psychiatric risk involved with these disorders [53] and the highly publicized harms when eating disorder chatbots have deviated from preprogrammed answers (eg, Tessa chatbot [30]). Potentially, the further use of ED ESSI in clinical settings will help to rebuild trust in patients and health professionals and pave the way for more advanced generative artificial intelligence agents to be introduced more broadly [31]. The positive results for ED ESSI are also another demonstration of a successful co-design process with key stakeholders, particularly those with lived eating disorder experience, leading to efficacious results with actual patients, where ED ESSI chatbot’s usability was rated as excellent [23,28,36]. Moreover, the results represent a further progression in the emerging field of SSIs for eating disorders [13,17,32,54]. Our chatbot SSI was broadly based on a psychologist-delivered SSI for adults seeking outpatient eating disorder treatment where the in-person SSI induced reductions in eating disorder symptoms (Cohen *d*=0.27), psychosocial impairment (Cohen *d*=0.29), and depression symptoms (Cohen *d*=0.21) as well as increased the likelihood of patients entering treatment (70%) [18]. As described in the Results section, this study’s chatbot SSI seemed to yield superior results in terms of effect sizes (Cohen *d*=0.35-0.53) for the same outcome measures and treatment uptake (28/30, 93%) as well as significant decreases in anxiety symptoms. Furthermore, Fursland et al [18] reported a significant increase in BMI units (0.9) for patients who were underweight (BMI<18.5 kg/m^2^) after SSI completion, which was not examined in this study for this cohort of patients and should be the subject of future research.

As proposed by Fursland et al [18], we suggest that the engaging psychoeducation and assessment delivered by the chatbot resulted in positive changes to patient attitudes and behaviors, and by delivering this information so soon after joining a waitlist, we were able to capitalize on the motivation for treatment. Indeed, there was an initial moderate to large increase in confidence in the ability to change immediately after using the chatbot, and although this was not maintained at subsequent time points, perhaps this was a particularly crucial time frame to start promoting positive changes in attitudes and behaviors. Furthermore, as found by Messer et al [16], there was no reduction in any of the moderate to high motivation measures for the chatbot (or control) group over the course of the study, so the SSI experience did not have negative impacts. Indeed, patients who are seeking treatment and are willing to be part of a research study where “extra” treatment is offered are likely to be highly motivated.

### Strengths, Limitations, and Future Research

The RCT was implemented in naturalistic settings in outpatient psychology clinics around Australia, and the attrition rate throughout the study was very low, particularly compared to other SSI studies in the same field of research (eg, [16]). The highest level of attrition observed in our study occurred at the screening survey stage rather than at any point after participants had completed the chatbot or control intervention. From our discussions with the clinics involved in the study, it is possible that patients may have mistaken our study screening survey web link for the clinic’s standard intake registration web link, with all information being sent in a single email or correspondence. Thus, individuals may have stopped completing the screening when they realized that it was part of a separate research study rather than their core treatment at the clinic. Another strength of our study and the chatbot’s design [36] was the ability to assess and alert when a user’s psychiatric and/or medical risk was detected. Although not experiencing high medical and/or psychiatric risk was an eligibility criterion for our RCT, this level of risk can readily change while waiting for and during the course of treatment [53]. The chatbot alert system was triggered for 1 participant, and follow-up with the research and clinical team was performed. The participant reported that they triggered the alert “by accident,” but it was, nevertheless, a demonstration of the alert system functioning appropriately in a naturalistic setting. Owing to the previous instances of malfunctioning chatbots in our field of eating disorders (eg, [30]), demonstrating no adverse events is especially important. A further strength of our study was that all participants involved were offered treatment by the 3-month postbaseline time point, and a proportion (29/60, 48%) of participants had started by 1 month. This not only allowed for the investigation of receptiveness to treatment and reasons for not commencing, which were primarily financial in nature, but also revealed loss of confidence in the ability to recover for some control group participants. Financial concerns were most likely reflective of the increase in the cost of living in Australia and globally [55] and potentially emphasize the importance of free or low-cost online eating disorder treatment offerings.

There are also limitations to this research that must be considered. First, we did not specifically examine whether the chatbot or control group engaged with the psychoeducation content through, for example, a knowledge check immediately after intervention. Furthermore, a more optimal control group should be used in future research. For example, the control participants could have been provided with a sham chatbot, which included engagement-related factors similar to ED ESSI chatbot and an active component that differed from ED ESSI chatbot’s psychoeducation and assessment approach, such as self-compassion [56]. Such a strategy will also likely assist ED ESSI with broader dissemination and regulatory approval internationally [56]. Furthermore, the participant sample consisted predominantly of White, younger cisgender women. This is reflective of the demographic characteristics of individuals who most often seek treatment [57]; however, there is great diversity in the people who experience eating disorders [58,59]. It will be important to examine the effectiveness of the chatbot in more diverse demographic samples. Potentially, ED ESSI may be even more appealing to diverse samples owing to high accessibility, convenience, and the fact that the chatbot has been explicitly programmed not to stigmatize the user, as has been unfortunately documented for some health professionals working in the field of eating disorders [60,61]. However, it will also be critical to explore other implementation strategies for ED ESSI in future research, for example, access via a website or social media chat functions in accordance with how we have previously used JEM in multiple countries [34,35]. Such strategies are possibly more likely to reach diverse users, as we found for JEM and should be investigated for ED ESSI. Furthermore, we did not have the statistical power to investigate the effectiveness of the chatbot by, for example, different eating disorder diagnoses or BMI categories. There was some diversity in these areas in the RCT, and future research would usefully focus on deciphering whether chatbot use is more optimal for certain clinical presentations and demographic characteristics. Moreover, it appeared that our participants were moderately to highly motivated at the commencement of the study, which may explain why they were receptive to participating in our research. It will be vital to determine the chatbot’s effectiveness in individuals experiencing lower levels of motivation. Finally, although a 3-month follow-up time is fairly standard in the SSI literature [15], a longer follow-up time would be beneficial in future research. It is possible that chatbot users required less time and fewer treatment sessions with a psychologist and other health professionals in order to reach recovery. However, it is also possible that those who did not use the chatbot “caught up” in terms of treatment gains after the 3-month time point and ended up at a similar phase of recovery in a similar amount of time.

### Conclusions

In conclusion, a novel chatbot-delivered SSI for older adolescents and adults on the waitlist for outpatient treatment for any type of eating disorder effectively reduced eating disorder pathology, psychosocial impairment, depression, and anxiety at 1 and 3 months in an RCT. Furthermore, chatbot usability was excellent, and users were more likely to enter in-person treatment when it became available. ED ESSI potentially represents an effective, accessible, convenient, and scalable form of early intervention for people waiting for eating disorder treatment; however, further research is required in more diverse samples and with longer follow-up periods.

## Appendix

Multimedia Appendix 1 Primary and secondary subscale outcomes for the chatbot (n=30) versus control (n=30) interventions over time point 1, time point 3, and time point 4.

Multimedia Appendix 2 CONSORT-eHEALTH checklist (V 1.6.1).

## Abbreviations

CBT: cognitive behavioral therapy
CBT-E: enhanced cognitive behavioral therapy
CIA: Clinical Impairment Assessment
DASS-21: Depression Anxiety Stress Scale-21
ED ESSI: Eating Disorder Electronic Single-Session Intervention
EDE-Q: Eating Disorder Examination Questionnaire
RCT: randomized controlled trial
SSI: single-session intervention
SUS: System Usability Scale
T1: time point 1
T2: time point 2
T3: time point 3
T4: time point 4

## Data Availability

The datasets generated and analyzed during this study are not publicly available under the participant confidentiality conditions of ethics approval from the Monash University Human Research Ethics Committee (ID 38277). The corresponding author can be contacted with queries about the data.

## References

[1] American Psychiatric Association (2022). Diagnostic and Statistical Manual Of Mental Disorders: DSM-5-TR, 5th edition, Text revision.

[2] Hay P, Aouad P, Le A, Marks P, Maloney D, Touyz S, Maguire S, National Eating Disorder Research Consortium (2023). Epidemiology of eating disorders: population, prevalence, disease burden and quality of life informing public policy in Australia-a rapid review. J Eat Disord.

[3] Allen KL, Mountford VA, Elwyn R, Flynn M, Fursland A, Obeid N, Partida G, Richards K, Schmidt U, Serpell L, Silverstein S, Wade T (2023). A framework for conceptualising early intervention for eating disorders. Eur Eat Disord Rev.

[4] Sukunesan S, Huynh M, Sharp G (2021). Examining the pro-eating disorders community on twitter via the hashtag #proana: statistical modeling approach. JMIR Ment Health.

[5] Treasure J, Russell G (2011). The case for early intervention in anorexia nervosa: theoretical exploration of maintaining factors. Br J Psychiatry.

[6] Arcelus J, Mitchell AJ, Wales J, Nielsen S (2011). Mortality rates in patients with anorexia nervosa and other eating disorders. A meta-analysis of 36 studies. Arch Gen Psychiatry.

[7] Austin A, Flynn M, Richards K, Hodsoll J, Duarte TA, Robinson P, Kelly J, Schmidt U (2021). Duration of untreated eating disorder and relationship to outcomes: a systematic review of the literature. Eur Eat Disord Rev.

[8] Fairburn CG, Harrison PJ (2003). Eating disorders. Lancet.

[9] Treasure J, Duarte TA, Schmidt U (2020). Eating disorders. Lancet.

[10] Byrne SM, Fursland A, Allen KL, Watson H (2011). The effectiveness of enhanced cognitive behavioural therapy for eating disorders: an open trial. Behav Res Ther.

[11] Carter O, Pannekoek L, Fursland A, Allen KL, Lampard AM, Byrne SM (2012). Increased wait-list time predicts dropout from outpatient enhanced cognitive behaviour therapy (CBT-E) for eating disorders. Behav Res Ther.

[12] Campbell A (2013). Single session interventions: an example of clinical research in practice. ANZ J of Family Therapy.

[13] Schleider JL, Smith AC, Ahuvia I (2023). Realizing the untapped promise of single-session interventions for eating disorders. Int J Eat Disord.

[14] Schleider JL, Dobias ML, Sung JY, Mullarkey MC (2020). Future directions in single-session youth mental health interventions. J Clin Child Adolesc Psychol.

[15] Schleider JL, Weisz JR (2017). Little treatments, promising effects? Meta-analysis of single-session interventions for youth psychiatric problems. J Am Acad Child Adolesc Psychiatry.

[16] Messer M, Fuller-Tyszkiewicz M, Liu C, Anderson C, Linardon J (2024). A randomized controlled trial of an online single session intervention for body image in individuals with recurrent binge eating. Int J Eat Disord.

[17] Wade TD (2023). Developing the "single-session mindset" in eating disorder research: commentary on Schleider et al., 2023 "Realizing the untapped promise of single-session interventions for eating disorders". Int J Eat Disord.

[18] Fursland A, Erceg-Hurn DM, Byrne SM, McEvoy PM (2018). A single session assessment and psychoeducational intervention for eating disorders: impact on treatment waitlists and eating disorder symptoms. Int J Eat Disord.

[19] Sung JY, Bugatti M, Vivian D, Schleider JL (2023). Evaluating a telehealth single-session consultation service for clients on psychotherapy wait-lists. Pract Innov.

[20] Haque MD, Rubya S (2023). An overview of chatbot-based mobile mental health apps: insights from app description and user reviews. JMIR Mhealth Uhealth.

[21] He Y, Yang L, Qian C, Li T, Su Z, Zhang Q, Hou X (2023). Conversational agent interventions for mental health problems: systematic review and meta-analysis of randomized controlled trials. J Med Internet Res.

[22] Vaidyam AN, Wisniewski H, Halamka JD, Kashavan MS, Torous JB (2019). Chatbots and conversational agents in mental health: a review of the psychiatric landscape. Can J Psychiatry.

[23] Beilharz F, Sukunesan S, Rossell SL, Kulkarni J, Sharp G (2021). Development of a positive body image chatbot (KIT) with young people and parents/carers: qualitative focus group study. J Med Internet Res.

[24] Høiland CG, Følstad A, Karahasanovic A (2020). Hi, can I help? Exploring how to design a mental health chatbot for youths. Hum Technol.

[25] Zhong W, Luo J, Zhang H (2024). The therapeutic effectiveness of artificial intelligence-based chatbots in alleviation of depressive and anxiety symptoms in short-course treatments: a systematic review and meta-analysis. J Affect Disord.

[26] Chan WW, Fitzsimmons-Craft EE, Smith AC, Firebaugh M, Fowler LA, DePietro B, Topooco N, Wilfley DE, Taylor CB, Jacobson NC (2022). The challenges in designing a prevention chatbot for eating disorders: observational study. JMIR Form Res.

[27] Matheson EL, Smith HG, Amaral AC, Meireles JF, Almeida MC, Linardon J, Fuller-Tyszkiewicz M, Diedrichs PC (2023). Using chatbot technology to improve Brazilian adolescents' body image and mental health at scale: randomized controlled trial. JMIR Mhealth Uhealth.

[28] Shah J, DePietro B, D'Adamo L, Firebaugh M, Laing O, Fowler LA, Smolar L, Sadeh-Sharvit S, Taylor CB, Wilfley DE, Fitzsimmons-Craft EE (2022). Development and usability testing of a chatbot to promote mental health services use among individuals with eating disorders following screening. Int J Eat Disord.

[29] Fitzsimmons-Craft EE, Chan WW, Smith AC, Firebaugh M, Fowler LA, Topooco N, DePietro B, Wilfley DE, Taylor CB, Jacobson NC (2022). Effectiveness of a chatbot for eating disorders prevention: a randomized clinical trial. Int J Eat Disord.

[30] Jargon J A chatbot was designed to help prevent eating disorders. Then it gave dieting tips. The Wall Street Journal.

[31] Sharp G, Torous J, West ML (2023). Ethical challenges in AI approaches to eating disorders. J Med Internet Res.

[32] Nemesure MD, Park C, Morris RR, Chan WW, Fitzsimmons-Craft EE, Rackoff GN, Fowler LA, Taylor CB, Jacobson NC (2023). Evaluating change in body image concerns following a single session digital intervention. Body Image.

[33] Fitzsimmons-Craft EE, Rackoff GN, Shah J, Strayhorn JC, D'Adamo L, DePietro B, Howe CP, Firebaugh M, Newman MG, Collins LM, Taylor CB, Wilfley DE (2024). Effects of chatbot components to facilitate mental health services use in individuals with eating disorders following online screening: an optimization randomized controlled trial. Int J Eat Disord.

[34] Navigating mental health with technology. Foundation UHN.

[35] Sharp G KIT the Chatbot’s first year of life: happy birthday KIT!. Butterfly Foundation.

[36] Sharp G, Dwyer B, Xie J, McNaney R, Shrestha P, Prawira C, Fernando AN, de Boer K, Hu H (2025). Co-design of a single session intervention chatbot for people on waitlists for eating disorder treatment: a qualitative interview and workshop study. J Eat Disord.

[37] Karkosz S, Szymański R, Sanna K, Michałowski J (2024). Effectiveness of a web-based and mobile therapy chatbot on anxiety and depressive symptoms in subclinical young adults: randomized controlled trial. JMIR Form Res.

[38] Faul F, Erdfelder E, Lang A, Buchner A (2007). G*Power 3: a flexible statistical power analysis program for the social, behavioral, and biomedical sciences. Behav Res Methods.

[39] Sharp G The impacts of a single session educational chatbot on eating disorder symptoms in people on waitlists for treatment. Australia New Zealand Clinical Trials Registry.

[40] Gatt L, Jan S, Mondraty N, Horsfield S, Hart S, Russell J, Laba TL, Essue B (2014). The household economic burden of eating disorders and adherence to treatment in Australia. BMC Psychiatry.

[41] Eating disorders. Centre for Clinical Interventions.

[42] Fairburn CG (2008). Cognitive Behavior Therapy and Eating Disorders.

[43] Eating disorder treatment and management plans. National Eating Disorders Collaboration.

[44] Bohn K, Doll HA, Cooper Z, O'Connor M, Palmer RL, Fairburn CG (2008). The measurement of impairment due to eating disorder psychopathology. Behav Res Ther.

[45] Lovibond SH, Lovibond PF (1995). Manual for the depression anxiety stress scales. 2nd edition. Psychology Foundation of Australia.

[46] Schmidt U, Startup H, Treasure J (2018). A Cognitive-Interpersonal Therapy Workbook for Treating Anorexia Nervosa: The Maudsley Model.

[47] Brooke J, Jordan PW, Thomas B, Weerdmeester BA, McClelland AL (1996). SUS: a 'quick and dirty' usability scale. Usability Evaluation in Industry.

[48] IBM SPSS Statistics for Windows, Version 29.0. IBM Corp.

[49] VanderWeele TJ, Mathur MB (2019). Some desirable properties of the Bonferroni correction: is the Bonferroni correction really so bad?. Am J Epidemiol.

[50] Cohen J (1992). A power primer. Psychol Bull.

[51] Bengtsson M (2016). How to plan and perform a qualitative study using content analysis. NursingPlus Open.

[52] Iacobucci G (2021). Eating disorders: record number of young people wait for treatment as demand soars. BMJ.

[53] Hay P, Chinn D, Forbes D, Madden S, Newton R, Sugenor L, Touyz S, Ward W, Royal Australian and New Zealand College of Psychiatrists (2014). Royal Australian and New Zealand college of psychiatrists clinical practice guidelines for the treatment of eating disorders. Aust N Z J Psychiatry.

[54] Smith AC, Ahuvia I, Ito S, Schleider JL (2023). Project body neutrality: piloting a digital single-session intervention for adolescent body image and depression. Int J Eat Disord.

[55] World economic outlook: policy pivot, rising threats. International Monetary Funds.

[56] Bos E, Preller KH, Kaur G, Malhotra P, Kharawala S, Motti D (2023). Challenges with the use of digital sham: systematic review and recommendations. J Med Internet Res.

[57] Field AE, Ziobrowski HN, Eddy KT, Sonneville KR, Richmond TK (2024). Who gets treated for an eating disorder? Implications for inference based on clinical populations. BMC Public Health.

[58] Qian J, Wu Y, Liu F, Zhu Y, Jin H, Zhang H, Wan Y, Li C, Yu D (2022). An update on the prevalence of eating disorders in the general population: a systematic review and meta-analysis. Eat Weight Disord.

[59] Santomauro DF, Melen S, Mitchison D, Vos T, Whiteford H, Ferrari AJ (2021). The hidden burden of eating disorders: an extension of estimates from the Global Burden of Disease Study 2019. Lancet Psychiatry.

[60] Lubieniecki G, Fernando AN, Randhawa A, Cowlishaw S, Sharp G (2024). Perceived clinician stigma and its impact on eating disorder treatment experiences: a systematic review of the lived experience literature. J Eat Disord.

[61] Sharp G, Fernando AN, Davis SR, Randhawa A (2024). Developing an educational resource for people experiencing eating disorders during the menopause transition: a qualitative co-design study. J Eat Disord.

