# Supplementary material for: The Effectiveness of a Chatbot Single-Session Intervention for People on Waitlists for Eating Disorder Treatment: Randomized Controlled Trial
Source: J Med Internet Res. 2025 May 21;27:e70874. doi: 10.2196/70874 (PMC12138310; doi:10.2196/70874)
Supplement: Multimedia Appendix 1 [file jmir_v27i1e70874_app1.docx]

**Supplementary Table 1.** Primary and Secondary Subscale Outcomes for the Chatbot (*n* = 30) versus Control Interventions (*n* = 30) over the T1, T3 and T4 timepoints.

|  | **Baseline (T1)**  **Mean (SD)** | **1 Month (T3)**  **Mean (SD)** | **3 Months (T4)**  **Mean (SD)** |
| --- | --- | --- | --- |
| **EDE-Q Subscales** | | | |
| **Dietary Restraint** | | | |
| Chatbot | 3.6 (1.6) | 3.2 (1.7) | 2.7 (1.5) |
| Control | 3.6 (1.4) | 3.6 (1.5) | 3.3 (1.3) |
| **Eating Concern** | | | |
| Chatbot | 3.4 (1.4) | 2.9 (1.4) | 2.8 (1.3) |
| Control | 3.4 (1.3) | 3.4 (1.3) | 3.3 (1.3) |
| **Shape Concern** | | | |
| Chatbot | 4.5 (1.2) | 4.0 (1.4) | 3.7 (1.5) |
| Control | 4.5 (1.2) | 4.5 (1.3) | 4.3 (1.2) |
| **Weight Concern** | | | |
| Chatbot | 4.3 (1.3) | 3.7 (1.4) | 3.3 (1.3) |
| Control | 4.4 (1.2) | 4.3 (1.2) | 4.1 (1.1) |
|  | | | |
| **CIA Subscales** | | | |
| **Cognitive Impairment** | | | |
| Chatbot | 10.2 (4.7) | 7.2 (4.0) | 5.5 (4.0) |
| Control | 10.7 (4.8) | 10.9 (4.3) | 9.4 (3.3) |
| **Personal Impairment** | | | |
| Chatbot | 14.1 (4.1) | 12.6 (4.5) | 11.5 (5.0) |
| Control | 14.4 (4.0) | 14.0 (3.7) | 12.3 (3.5) |
| **Social Impairment** | | | |
| Chatbot | 9.3 (4.1) | 8.1 (4.6) | 6.6 (4.5) |
| Control | 9.6 (4.2) | 9.0 (3.8) | 7.8 (2.9) |
